# Nonstoichiometric LaO_0.65_F_1.7_ Structure and Its Green Luminescence Property Doped with Bi^3+^ and Tb^3+^ Ions for Applying White UV LEDs

**DOI:** 10.3390/ma15124222

**Published:** 2022-06-14

**Authors:** Sungjun Yang, Seungyong Shin, Heonji Ha, Sangmoon Park

**Affiliations:** 1 Department of Environmental Energy and Chemistry, College of Engineering, Silla University, Busan 46958, Korea; qse7417@naver.com (S.Y.); ljhbb0211@naver.com (S.S.); hhji1028@naver.com (H.H.); 2 Department of Fire Protection and Safety Management, College of Health and Welfare, Silla University, Busan 46958, Korea

**Keywords:** X-ray diffraction, phosphors, Bi^3+^, Tb^3+^ energy transfer, pc LED

## Abstract

Red–green–blue phosphors excited by ultraviolet (UV) radiation for white light LEDs have received much attention to improve the efficiency, color rendering index (CRI), and chromatic stability. The spectral conversion of a rare-earth ion-doped nonstoichiometric LaO_0.65_F_1.7_ host was explored with structural analysis in this report. The nonstoichiometric structure of a LaO_0.65_F_1.7_ compound, synthesized by a solid-state reaction using La_2_O_3_ and excess NH_4_F precursors, was analyzed by synchrotron X-ray powder diffraction. The crystallized LaO_0.65_F_1.7_ host, which had a tetragonal space group of *P4/nmm*, contained 9- and 10-coordinated La^3+^ sites. Optical materials composed of La_1−*p*−_*_q_*Bi*_p_*Tb*_q_*O_0.65_F_1.7_ (*p* = 0 and 0.01; *q* = 0–0.2) were prepared at 1050 °C for 2 h, and the single phase of the obtained phosphors was indexed by X-ray diffraction analysis. The photoluminescence spectra of the energy transfer from Bi^3+^ to Tb^3+^ were obtained upon excitation at 286 nm in the nonstoichiometric host lattice. The desired Commission Internationale de l’Eclairage (CIE) values of the phosphors were calculated. The intense green La_0.89_Bi_0.01_Tb_0.1_O_0.65_F_1.7_ phosphor with blue and red optical materials was fabricated on a 275 nm UV-LED chip, resulting in white light, and the internal quantum efficiency, CRI, correlated color temperature, and CIE of the pc LED were characterized.

## 1. Introduction

Phosphor-converted light-emitting diodes (pc LEDs) have been developed as common light sources for various lighting and display industries [1,2,3,4,5]. Yellow garnet phosphors, such as Ce^3+^-doped Y_3_Al_5_O_12_ compounds, fabricated on blue LED chips are widely used to generate white light sources. LEDs have excellent advantages, such as low cost and easy fabrication; however, they have several weaknesses such as relatively low efficiency, color rendering index (CRI), and chromatic stability [1,2,3,4,5]. Meanwhile, red–green–blue (RGB) pc LEDs excited by ultraviolet (UV) radiation have strengths, such as higher efficiency and a CRI with higher chromatic stability when subjected to different driving currents, compared with blue-excitable pc LEDs [1,2,3,4,5]. Although RGB tri-color phosphors are intricately blended for fabrication on an LED chip, specific mixtures of RGB phosphors can result in tunable color temperatures. When excited by UV radiation in various host lattices, Bi^3+^ ions can emit radiation in the blue-to-green wavelength regions associated with 6*s*^1^6*p*^1^ to 6*s*^2^ transitions [6,7,8,9,10]. The Bi^3+^ ion, behaving as a donor, can facilitate energy transfer and improve the emission of light from acceptors in the host structures such as Eu^3+^ or Tb^3+^ ions [6,7,8,9,10]. The Bi^3+^ and Eu^3+^ codoped nonstoichiometric LaO_0.65_F_1.7_ phosphor was utilized, as a red emitter, under UV excitation in a previous study [6]. The nonstoichiometric LaO_0.65_F_1.7_ host has advantageous optical qualities when compared with stoichiometric LaOF compounds [6,11,12]. Rare-earth ion-doped nonstoichiometric hosts show a broad excitation range with stronger Stark splitting. Furthermore, they have better spectral conversion properties owing to their low nonradiative relaxation caused by the low phonon frequency [6,11,12]. The LaO_0.65_F_1.7_ lattice, which is stacked by 9-coordinated LaO_2_F_7_ and 10-coordinated LaO_3_F_7_ layers along the *c*-axis with the tetragonal space group *P4/nmm*, was studied by high-resolution powder neutron diffraction analysis [13]. The single phase of the LaO_0.65_F_1.7_ host was synthesized using a high temperature and long reaction period at 1200 °C for 2 d in a nickel-sealed tube [13]. In this study, a LaO_0.65_F_1.7_ host was prepared using the flux method at 1050 °C for 2 h in air [6,11]. The nonstoichiometric LaO_0.65_F_1.7_ structure was refined using synchrotron powder X-ray diffraction data at room temperature. The luminescence spectra of the La_1−*p*−*q*_Bi*_p_*Tb*_q_*O_0.65_F_1.7_ (*p* = 0 and 0.01; *q* = 0–0.2) phosphors were explored by examining the energy-transfer mechanism from Bi^3+^ to Tb^3+^. UV-excitable white light was emitted using a green La_0.96_Bi_0.01_Tb_0.02_Eu_0.01_O_0.65_F_1.7_ phosphor with blue and red phosphors on a 275 nm UV-LED chip. The internal quantum efficiency (IQE), CRI, correlated color temperature (CCT), and Commission Internationale de l’Eclairage (CIE) coordinates of the RGB pc LEDs were calculated.

## 2. Experimental Section

Synchrotron powder X-ray diffraction data of the nonstoichiometric LaO_0.65_F_1.7_ host were collected at the PLS-II 6D UNIST-PAL beamline of the Pohang Accelerator Laboratory (PAL). The powdered sample was loaded into a quartz capillary (diameter: 200 μm) and rotated during the data collection to eliminate the preferred orientation effect. Monochromatic X-rays (λ = 0.65303 Å, 18.986 keV) and a charge-coupled device detector (MX225-HS, Rayonix, Evanston, IL, USA) were used in these experiments [14]. The LaO_0.65_F_1.7_ structure was refined using the Rietveld refinement program FullProf Suite [15,16]. Phosphors of La_1−*p*−*q*_Bi*_p_*Tb*_q_*O_0.65_F_1.7_ (*p* = 0 and 0.01; *q* = 0–0.2) were prepared by heating the appropriate stoichiometric molar amounts of La_2_O_3_ (Alfa 99.9%), Bi_2_O_3_ (Alfa 99.99%), and Tb_4_O_7_ (Alfa 99.9%) with excess NH_4_F (Alfa 99%) precursor [6,11]. Powdered samples with 1:2 molar ratios of La(Bi,Tb)O_3/2_ and NH_4_F were used to prepare the LaO_0.65_F_1.7_:Bi^3+^ and Tb^3+^ phosphors. The La(Bi,Tb)O_3/2_ and NH_4_F precursors were mixed using an agate mortar and pestle and, subsequently, heated at 1050 °C for 2 h in air [6,11]. The La_2_O_3_ precursor was used after preheating at 700 °C for 3 h to remove hydroxide from the acquired sample. The phase identification of the La_1−*p*−*q*_Bi*_p_*Tb*_q_*O_0.65_F_1.7_ phosphors was performed using a Shimadzu XRD-6000 powder diffractometer (Cu-*Kα* radiation). The excitation and emission photoluminescent spectra of the phosphors were measured using a spectrofluorometer (Sinco Fluoromate FS-2, Seoul, Korea). The pc LED was fabricated by packing the green La_0.96_Bi_0.01_Tb_0.02_Eu_0.01_O_0.65_F_1.7_, blue Ba_4_Ca_5_Al_2_Si_6_O_24_:Ce,Na, and red LaO_0.65_F_1.7_:Bi,Eu phosphors in-between quartz glasses on a 275 nm LED chip (Seoul Semiconductor, Ansan-si, Korea). A spectrometer (USB4000, Ocean Optics, Dunedin, FL, USA) was used to measure the CRI, CCT, CIE coordinates, and IQE of the white-light pc LEDs.

## 3. Results and Discussion

**Figure 1:**
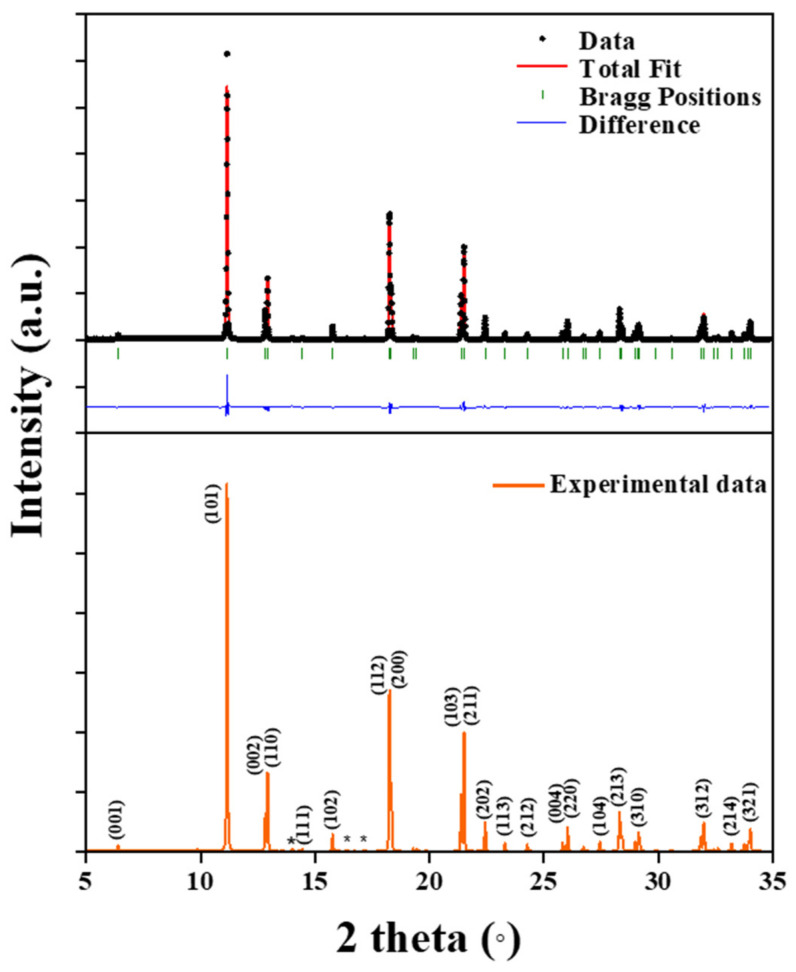
Synchrotron XRD pattern of a tetragonal LaO_0.65_F_1.7_ sample.

Rietveld refinement fitting of the synchrotron powder X-ray diffraction data of the nonstoichiometric LaO_0.65_F_1.7_ structure at room temperature is shown in Figure 1. Unidentified small impurities at approximately 14° and between 15 and 18°, which are marked, were observed in the experimental XRD data. The refinement and crystal data are presented in Table 1. The nonstoichiometric LaO_0.65_F_1.7_ host crystallized in the tetragonal space group *P4/nmm*, which has *a* and *c* cell parameters of *a* = 4.10058 (6) Å and *c* = 5.8468 (1) Å. The refined atomic coordinates, equivalent isotropic displacement parameters with Wyckoff positions, and site occupation factors are listed in Table 2. 

**Figure 2:**
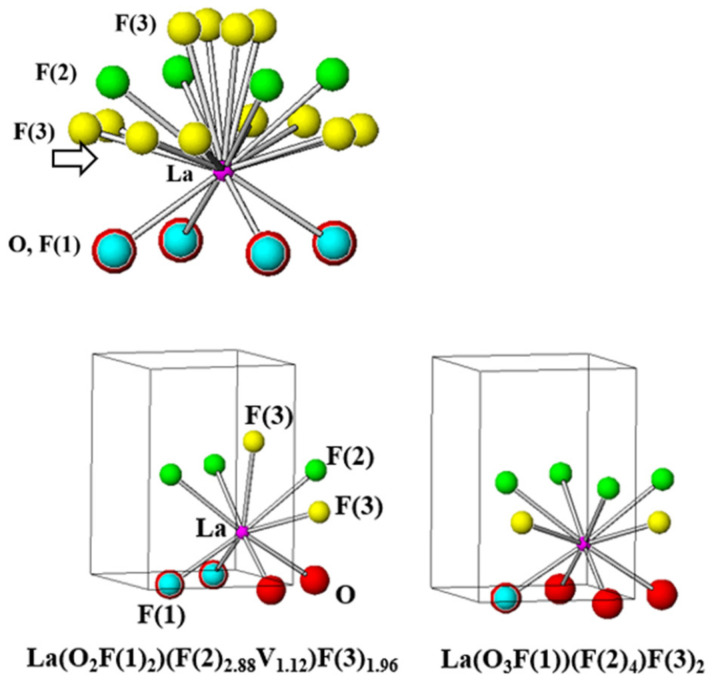
Polyhedrons of LaO_0.65_F_1.7_, 9-coordinated La(O_2_F(1)_2_)(F(2)_2.88_V_1.12_)F(3)_1.96_, and 10-coordinated La(O_3_F(1))(F(2)_4_)F(3)_2_ structures.

The formula of the LaO_0.65_F_1.7_ structure can be expressed as LaO_0.65_F(1)_0.35_F(2)_0.86_F(3)_0.49_ based on the calculations of the site occupancies and Wyckoff positions in the unit cell. All possible bonds between the La^3+^ cations and O^2−^/F^−^ anions in the host lattice from the crystal data contain La-O_4_, La-F(1)_4_, La-F(2)_4_, and La-F(3)_12_ as shown in Figure 2 and Table 3. 

In La-F(3)_12_, there were four long and eight short La-F(3) bond distances of 2.591 (18) and 2.549 (12) Å, respectively. The polyhedron based on the nonstoichiometric LaO_0.65_F(1)_0.35_F(2)_0.86_F(3)_0.49_ structure refinement can be represented as La(O_0.65_F(1)_0.35_)(F(2)_0.86_V_0.14_)(F(3)_0.49_), which contains a vacancy (V) associated with the F(2) anion. In a previous study, the site dependency of 9- and 10-coordinated La^3+^ sites in Eu^3+^-doped LaO_0.65_F_1.7_ phosphors was reported [6,13]. When Eu^3+^ ions were substituted in the LaO_0.65_F_1.7_ structure, both 9-coordinated no-inversion and 10-coordinated symmetric inversion sites were observed from the Eu^3+^ transitions of the emission spectra, which showed the ^5^D_0_–^7^F_2_ electric-dipole and ^5^D_0_–^7^F_1_ magnetic dipole transitions, respectively [6]. The polyhedrons in the LaO_0.65_F_1.7_ structure can be distinguished as both 9-coordinated La(O_2_F(1)_2_)(F(2)_2.88_V_1.12_)F(3)_1.96_ and 10-coordinated La(O_3_F(1))(F(2)_4_)F(3)_2_ as shown in Figure 2 [6,13]. 

**Figure 3:**
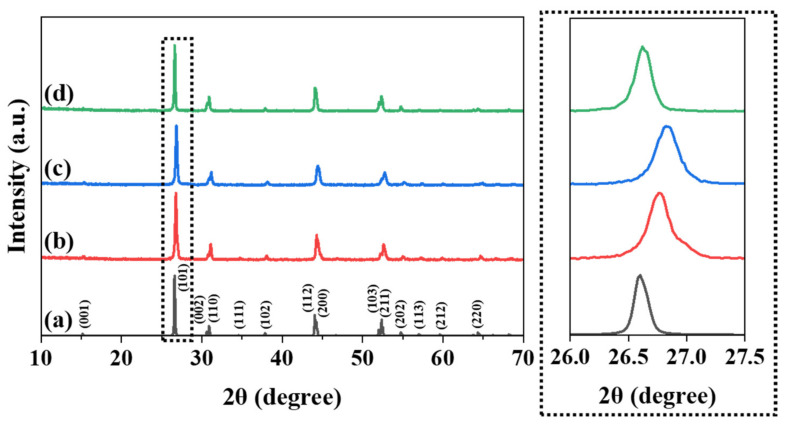
The calculated XRD patterns of (**a**) LaO_0.65_F_1.7_ (ICSD 40371) and the obtained XRD patterns of La_1−*p*−*q*_Bi*_p_*Tb*_q_*O_0.65_F_1.7_ phosphors (**b**) *q* = 0.01; (**c**) *q* = 0.1; (**d**) *p* = 0.01 and *q* = 0.1.

The crystallographic phase of the La_1−*p*−*q*_Bi*_p_*Tb*_q_*O_0.65_F_1.7_ (*p* = 0 and 0.01; *q* = 0–0.2) phosphors was identified using powder X-ray diffraction (XRD) patterns. The calculated XRD pattern of the tetragonal LaO_0.65_F_1.7_ (ICSD 40371) structure is shown in Figure 3a [13]. Figure 3b–d show the XRD patterns of the nonstoichiometric La_1−*p*−*q*_Bi*_p_*Tb*_q_*O_0.65_F_1.7_ phosphors (*q* = 0.01; *q* = 0.1; *p* = 0.01; *q* = 0.1, respectively). The XRD patterns of the obtained phosphors show a single phase, without any visible impurities, indexed to a tetragonal LaO_0.65_F_1.7_ structure. The Tb^3+^ activator can be located in the 9- and 10-coordinated La^3+^ sites of the nonstoichiometric LaO_0.65_F_1.7_ structure. When small Tb^3+^ ions (*r* = 1.04 Å for an eight coordination number (CN); *r* = 1.095 Å for a nine CN) were substituted for large La^3+^ ions (*r* = 1.16 Å for a a 8 CN; *r* = 1.216 Å for a 9 CN; *r* = 1.27 Å for a 10 CN) in the LaO_0.65_F_1.7_ host lattice, gradual shifts in the positions of the various Bragg reflections to higher angles were observed as shown in Figure 3b,c. The unit cell contraction of the cell parameters in the La_0.99_Tb_0.01_O_0.65_F_1.7_ and La_0.9_Tb_0.1_O_0.65_F_1.7_ phosphors occurred from a = 4.0749 (4) and c = 5.8188 (8) Å to a = 4.0689 (3) and c = 5.8057 (7) Å. Meanwhile, when Bi^3+^ ions (*r* = 1.17 Å for an eight CN) were substituted for La^3+^ ions in the La_0.9_Tb_0.1_O_0.65_F_1.7_ lattice, slight shifts in the positions of the various Bragg reflections to lower angles from the La_0.89_Bi_0.01_Tb_0.1_O_0.65_F_1.7_ phosphors (a = 4.0878 (1) and c = 5.8254 (3) Å) were observed as shown in Figure 3c,d, respectively.

**Figure 4:**
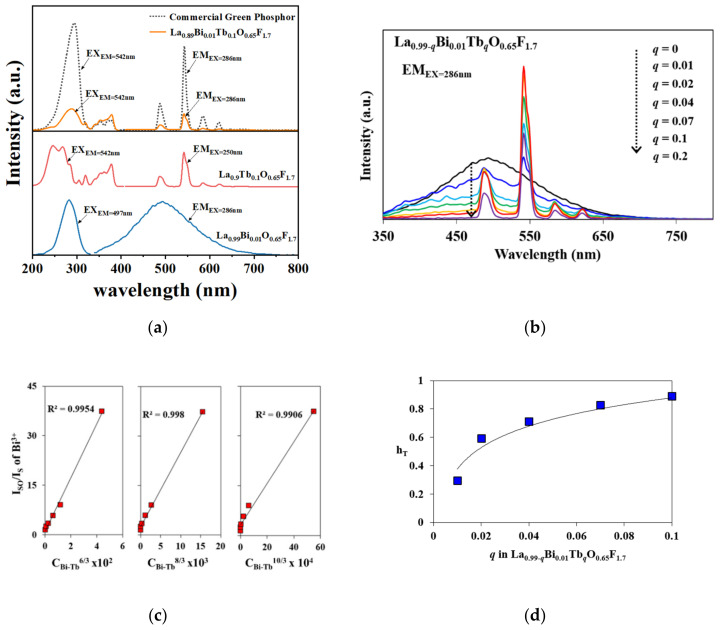
(**a**) PL excitation and emission spectra of La_0.99_Bi_0.01_O_0.65_F_1.7_, La_0.9_Tb_0.1_O_0.65_F_1.7_, La_0.89_Bi_0.01_Tb_0.1_O_0.65_F_1.7_, and commercial green LaPO_4_:Ce^3+^/Tb^3+^ phosphors; (**b**) the emission spectra of La_0.99−*q*_Bi_0.01_Eu*_q_*O_0.65_F_1.7_ (*q* = 0–0.2) phosphors under 286 nm excitation; (**c**) the plot of I_SO_/I_S_ versus C_Bi-Tb_^α/3^ (α = 6, 8, and 10); (**d**) energy-transfer efficiency from Bi^3+^ to Tb^3+^ in the phosphors.

Figure 4a shows the excitation and emission spectra of La_0.99_Bi_0.01_O_0.65_F_1.7_, La_0.9_Tb_0.1_O_0.65_F_1.7_, and La_0.89_Bi_0.01_Tb_0.1_O_0.65_F_1.7_ phosphors [6]. Previously, the photoluminescence properties of Bi^3+^-doped LaO_0.65_F_1.7_ phosphors were explored. The energy levels of the Bi^3+^ ions comprise ^1^S_0_, ^3^P_J_ (J = 0, 1, or 2), and ^1^P_1_ states. It is known that the ^1^S_0_ → ^3^P_1_ and ^1^P_1_ transitions arise from spin-orbital coupling, but the ^1^S_0_ → ^3^P_0_ and ^3^P_2_ transitions are forbidden [6,7,8,9,10]. The blue-emitting spectra of the Bi^3+^-doped LaO_0.65_F_1.7_ phosphors, attributed to the ^3^P_1_ → ^1^S_0_ transitions of the Bi^3+^ ions, revealed the emission band range from 350 to 650 nm under UV excitation as shown in Figure 4a. The maximum emission intensity was observed in a previous study when the Bi^3+^ concentration in the host lattice was 1 mol% [6]. The La_0.9_Tb_0.1_O_0.65_F_1.7_ phosphor was monitored from 400 to 700 nm while under UV excitation. The intense green emission of ^5^D_4_–^7^F_5_ transition from the Tb^3+^ ions was observed near 542 nm as shown in Figure 4a. The individual transitions of Bi^3+^ and Tb^3+^ ions, with energy transfer from Bi^3+^ to Tb^3+^ ions in the La_0.99−*q*_Bi_0.01_Tb*_q_*O_0.65_F_1.7_ (*q* = 0–0.2) phosphors, occurred under an excitation wavelength of 286 nm as shown in Figure 4b. The energy transfer from Bi^3+^ to Tb^3+^ operated as a sensitizer and activator, respectively, in the La_0.99−*q*_Bi_0.01_Tb*_q_*O_0.65_F_1.7_ (*q* = 0–0.2) phosphors. The emission of Tb^3+^ transitions was maximized when the Tb^3+^ content in the La_0.99−*q*_Bi_0.01_Tb*_q_*O_0.65_F_1.7_ (*q* = 0–0.2) phosphors was *q* = 0.1, which was compared with commercially available green LaPO_4_:Ce^3+^/Tb^3+^ phosphor in Figure 4a. The green emission of the La_0.89_Bi_0.01_Tb_0.1_O_0.65_F_1.7_ phosphor under 286 nm excitation significantly increased by ˃4 times compared to that of the La_0.9_Tb_0.1_O_0.65_F_1.7_ phosphor because of the energy transfer from Bi^3+^ to Tb^3+^ ions as shown in Figure 4b. The energy-transfer efficiency (η_T_) was estimated using the following formula:

η_T_ = 1 − I_S_/I_SO_

where I_S_ and I_SO_ are the luminescence intensities of the Bi^3+^ sensitizer in the presence and absence of a Tb^3+^ activator, respectively [6,7,8,9,10,17,18,19,20,21]. The energy-transfer mechanism can be represented by linear plots of I_SO_/I_S_ versus C_Bi-Tb_^α/3^, where C_Bi-Tb_ is the concentration of Bi^3+^ and Tb^3+^ ions, with α = 6, 8, or 10, corresponding to dipole–dipole, dipole–quadrupole, and quadrupole–quadrupole interactions, respectively, in accordance with the Dexter theory [17,18,19,20,21,22]. In Figure 4c, when α = 6, 8, and 10, the linear plots showed energy transfers from the Bi^3+^ to Tb^3+^ ions in the La_0.99−*q*_Bi_0.01_Tb*_q_*O_0.65_F_1.7_ (*q* = 0–0.2) phosphors with an R^2^ = 0.9954, 0.998, and 0.9906, respectively. When the value of α was 8, a closer linear plot was determined for the phosphor, and the dipole–quadrupole interaction was involved in the energy-transfer mechanism of the phosphors. The differences on the R-factors between dipole–dipole and dipole–quadrupole mechanisms are quite small when the concentrations of C_Bi-Tb_ and C_Tb_ were selected (Supplementary Materials, Figure S1). Possibly, there are some arguments that determine the energy-transfer process as the dipole–quadrupole interaction mechanism in the phosphors, because the concentration quenching selectively depends on whether it is a donor–donor or donor–acceptor for C^8/3^ concentration [23]. The efficiency gradually enhanced from 30 to 89% as the Tb^3+^ content in the phosphors increased from *q* = 0.01 to 0.1 as shown in Figure 4d. 

**Figure 5:**
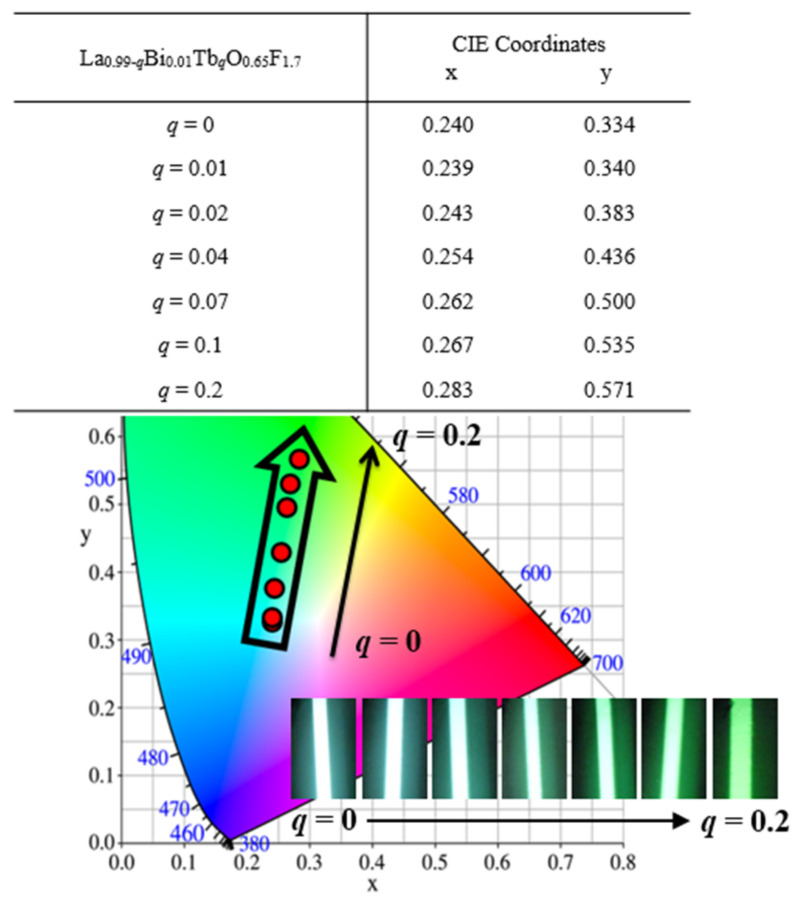
The chromaticity coordinates with the desired CIE values of La_0.99−*q*_Bi_0.01_Tb*_q_*O_0.65_F_1.7_ (*q* = 0–0.2) phosphors (EX = 286 nm) and photographs of the green emission light of the phosphors under 286 nm radiation.

**Figure 6:**
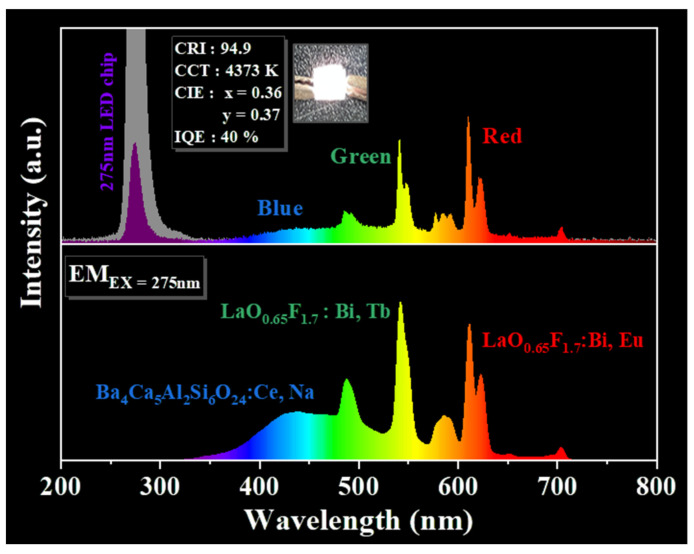
The emission spectra of RGB phosphors (La_0.94_Bi_0.01_Eu_0.05_O_0.65_F_1.7_, La_0.89_Bi_0.01_Tb_0.1_O_0.65_F_1.7_, and Ba_3.8_Ce_0.1_Na_0.1_Ca_5_Al_2_Si_6_O_24_) under 275 nm UV excitation and the electroluminescence spectra and a photograph of the RGB pc LED under 3.2 V and 20 mA.

As shown in Figure 5, the chromaticity coordinates, *x* and *y*, were in accordance with the desired CIE values from the blue to green wavelength regions for La_0.99−*q*_Bi_0.01_Tb*_q_*O_0.65_F_1.7_ (*q* = 0–0.2) phosphors (EX = 286 nm). When the concentration of Tb^3+^ ions in the La_0.99−*q*_Bi_0.01_Tb*_q_*O_0.65_F_1.7_ (*q* = 0–0.2) phosphors increased from *q* = 0 to 0.02 and 0.1, the emission colors exhibited a gradual shift from blue to green emission regions. The CIE values are summarized in the inset of Figure 5, along with the values obtained for the phosphors. The CIE coordinates of the blue and green regions of the phosphor CIE diagram were observed to be *x* = 0.240 and *y* = 0.334, *x* = 0.239 and *y* = 0.340, *x* = 0.254 and *y* = 0.436, and *x* = 0.267 and *y* = 0.535 for values of *q* = 0, 0.01, 0.04, and 0.1, respectively. The emission of the phosphors subject to 312 nm hand-lamp excitation was blue and green. This green-emitting light was adopted for a high color-rendering index to apply to pc UV LEDs. This La_0.89_Bi_0.01_Tb_0.1_O_0.65_F_1.7_ phosphor can be prepared as a green-emitting component for fabrication of a 275 nm UV LED chip. The photoluminescence and electroluminescence (EL) spectra resulting from 275 nm UV excitation of the green La_0.89_Bi_0.01_Tb_0.1_O_0.65_F_1.7_ phosphor with blue Ba_3.8_Ce_0.1_Na_0.1_Ca_5_Al_2_Si_6_O_24_ and red La_0.94_Bi_0.01_Eu_0.05_O_0.65_F_1.7_ phosphors were monitored as shown in Figure 6, respectively [8,24]. The pc UV LED was prepared at 3.2 V and 20 mA after the phosphors were packaged on a 275 nm LED chip (inset). A CRI (R_a_) of 94.9 at CCTs of 4373 K with CIE coordinates of *x* = 0.36 and *y* = 0.37 was determined for the 275 nm chip fabricated with the white-light pc UV LED. The three phosphors could generate white light in the 275 nm UV-executable LED chip. The IQE of the phosphors is expressed as η_QE_ using the following equation [25,26,27,28]:

η_QE_ = ∫L_S_/(∫E_R_ − ∫E_S_)

where L_S_, E_S_, and E_R_ are the luminescence integrated emission, excitation spectra of the phosphors, and the integrated excitation spectrum without phosphors in the sphere, respectively. The IQE of the white pc UV LED was approximately 40% under a 275 nm excitation. Color-tunable red, green, and blue phosphors can be diversely fabricated into UV-LED chips, which is an advantage for UV-executable LED applications.

## 4. Conclusions

The nonstoichiometric LaO_0.65_F_1.7_ structure was determined as a tetragonal unit cell (*P4/nmm*) with the cell parameters *a* = 4.10058 (6) Å and *c* = 5.8468 (1) Å using synchrotron X-ray powder diffraction. The LaO_0.65_F_1.7_ host lattice contained 9- and 10-coordinated La^3+^ sites in the 2c Wyckoff position. Optical materials composed of La_1−*p*−_*_q_*Bi*_p_*Tb*_q_*O_0.65_F_1.7_ (*p* = 0 and 0.01; *q* = 0–0.2) exhibited photoluminescence spectra characteristic of an energy transfer from Bi^3+^ to Tb^3^^+^ upon excitation with 286 nm involving dipole–quadrupole interactions in the phosphors. The desired Commission Internationale de l’Eclairage values of the blue–green phosphors were calculated. The green La_0.96_Bi_0.01_Tb_0.02_Eu_0.01_O_0.65_F_1.7_ phosphor was fabricated with blue and red phosphors on a 275 nm UV-LED chip, resulting in white light. The IQE was determined to be approximately 40% under a 275 nm excitation with a CRI (Ra) of 94.9 at CCT of 4373 K and CIE coordinates of *x* = 0.36 and *y* = 0.37.

## Figures and Tables

**Table 1:** Rietveld refinement and crystal data of a LaO_0.65_F_1.7_ structure.

Chemical Formula	LaO_0.65_F_1.7_
Radiation type, λ (Å)	Synchrotron (6D-BM), 0.65303
2θ range (deg)	5–35
Crystal system	Tetragonal
Space group	P 4/*n m m*
Lattice parameter (Å)	*a* = 4.10058 (6)
	c = 5.8468 (1)
Volume (Å^3^)	V = 98.313 (1)
R*_p_*	6.11
R*_wp_*	9.06
R*_exp_*	4.9
*S*	1.8
χ^2^	3.42

**Table 2:** Refined atomic coordinates and equivalent isotropic displacement parameters of LaO_0.65_F_1.7_.

Atom	Wyckoff Position	*x*	*y*	*z*	B_iso_	SOF
La	2*c*	0.25	0.25	0.2268 (3)	1.02 (6)	1
O	2*a*	0.75	0.25	0.00	1.1 (5)	0.65
F(1)	2*a*	0.75	0.25	0.00	1.1 (5)	0.35
F(2)	2*b*	0.75	0.25	0.50	2.6 (5)	0.86
F(3)	8*i*	0.25	0.077 (15)	0.652 (10)	4.8 (25)	0.1225

**Table 3:** Selected interatomic distances for LaO_0.65_F_1.7_.

Atom	Distance (Å)
La-O (x4)	2.4418 (3)
La-F(1) (x4)	2.4418 (3)
La-F(2) (x4)	2.5990 (4)
La-F(3) (x4)	2.591 (18)
La-F(3) (x8)	2.549 (12)

## Data Availability

The data presented in this study are available on request from the corresponding author.

## Supplementary Materials

The following supporting information can be downloaded at: https://www.mdpi.com/article/10.3390/ma15124222/s1, Figure S1. The plot of ISO/IS versus CTba/3 (a = 6, 8, 10).

## References

[1] Ye S., Xiao F., Parn Y.X., Ma Y.Y., Zhang Q.Y. (2010). Phosphors in phosphor-converted white light-emitting diodes: Recent advances in materials, techniques and properties. Mater. Sci. Eng. R.

[2] Cao L., Li W., Devakumar B., Ma N., Huang X., Lee A.F. (2022). Full-Spectrum White Light-Emitting Diodes Enabled by an Efficient Broadband Green-Emitting CaY_2_ZrScAl_3_O1_2_:Ce^3+^ Garnet Phosphor. ACS Appl. Mater. Interfaces.

[3] Ahn Y.N., Kim K.D., Anoop G., Kim G.S., Yoo J.S. (2019). Design of highly efficient phosphor converted white light-emitting diodes with color rendering indices (R_1_ − R_15_) ≥ 95 for artificial lighting. Sci. Rep..

[4] Fang M.H., Ni C., Zhang X., Tsai Y.T., Mahlik S., Lazarowska A., Grinberg M., Sheu H.S., Lee J.F., Cheng B.M. (2016). Enhance color rendering index via full spectrum employing the important key of cyan phosphor. ACS Appl. Mater. Interfaces.

[5] Schubert E.F., Kim J.K. (2005). Solid-state light sources getting smart. Science.

[6] Yang S., Park S. (2020). Bi^3+^ and Eu^3+^ Activated Luminescent Behaviors in Non-Stoichiometric LaO_0.65_F_1.7_ Structure. Materials.

[7] Ning H., Tian L. (2021). Enhanced green luminescence in BaZn_1.06_Al_9.94_O_17_:Tb^3+^ by co-doping with Bi^3+^ and energy transfer from Bi^3+^ to Tb^3+^. Optik.

[8] Taikar D.R. (2020). Study of energy transfer from Bi^3+^ to Tb^3+^ in Y_2_O_3_ phosphor and its application for W-LED. J. Alloys Compd..

[9] Tian S., Zhao L., Chen W., Liu Z., Fan X., Min Q., Yu H., Yu X., Qiu J., Xu X. (2018). Abnormal photo-stimulated luminescence in Ba_2_Ga_2_GeO_7_: Tb^3+^, Bi^3+^. J. Lumin..

[10] Yadav R.S., Rai S.B. (2017). Surface analysis and enhanced photoluminescence via Bi^3+^ doping in a Tb^3+^ doped Y_2_O_3_ nano-phosphor under UV excitation. J. Alloys Compd..

[11] Noh W., Park S. (2017). Synthesis and distinct up-converting behaviors of Er^3+^, Yb^3+^ doped LaOF and LaO_0.65_F_1.7_ phosphors. Opt. Mater..

[12] Yang W., Park S. (2016). Predominant green emission of Ce^3+^–Tb^3+^ activated Y_7_O_6_F_9_ phosphors. RSC Adv..

[13] Laval J.-P., Abaouz A., Frit B. (1988). High resolution powder neutron diffraction study of the tetragonal anion-excess fluorite-related LaF_1.70_O_0.65_ phase. Eur. J. Solid State Inorg. Chem..

[14] Shin S., Yanga S., Lee S.-H., Shin T.J., Park S. (2021). Distinctive occurrences of green-yellow luminescence from orthogermanate-type Ba_9_Y_2_(GeO_4_)_6_:Ce^3+^,Na^+^ phosphors under blue excitation and white-light performance with light-emitting diodes. J. Alloys Compd..

[15] Rodríguez-Carvajal J., Roisnel T. FullProf.98 and WinPLOTR New Windows Applications for Diffraction. Commission on Powder Diffraction. IUCr, Newsletter 20, May–August, 1998.

[16] Rodríguez-Carvajal J. Recent developments of the program FullProf”. Commission on Powder Diffraction. IUCr, Newsletter 26, December, 2001.

[17] Dexter D.L., Schulman J.H. (1954). Theory of concentration quenching in inorganic phosphors. J. Chem. Phys..

[18] Li K., Fan J., Shang M., Lian H., Lin J. (2015). Sr_2_Y_8_(SiO_4_)_6_O_2_:Bi^3+^/Eu^3+^: A single-component white-emitting phosphor via energy transfer for UV w-LEDs. J. Mater. Chem. C.

[19] Yang S., Kim H., Park S. (2018). Color-tunable luminescence in Y_7(1-m-n-z)_Bi_7m_Dy_7n_Eu_7z_O_6_F_9_ (m = 0.001-0.05, n = 0–0.1, z = 0.005, 0.01) phosphors. Opt. Mater..

[20] Yun H., Kim S.-H., Park S. (2017). Bi^3+^, Eu^3+^-doped Ba_9_Y_2_Si_6_O_24_ phosphors based on the site-selected substitution. Opt. Mater..

[21] Zou Y., Min X., Liu Z., Yu L., Liu B. (2020). Photoluminescent properties and energy transfer mechanism of Tb^3+^-Ce^3+^ doped CaSi_2_O_2_N_2_ oxynitride phosphors. Mater. Res. Bull..

[22] Xia M., Zhao W., Zhong J., Shi P., Liao Z., Liu X., Song J., Luo L., Ma L., Nie Z. (2020). Tunable luminescence of blue-green emitting NaBaBO_3_:Ce^3+^,Tb^3+^ phosphors for near-UV light emitting diodes. J. Lumin..

[23] Sontakke A.D., van Bunningen A.J., Rabouw F.T., Meijers S., Meijerink A. (2020). Unraveling the Eu^2+^ → Mn^2+^ Energy Transfer Mechanism in w-LED Phosphors. J. Phys. Chem. C.

[24] Yang S., Park S. (2020). Ca^2+^-substitution effects in Ba_9−x_Ca_x_Al_2_Si_6_O_24_:Ce^3+^,Na^+^ phosphors. Opt. Mater..

[25] Yang S., Park S. (2021). Luminescent performances of Ba_9−p_Ca_p_Al_2_Si_6_O_24_:Eu^2+^,Mn^2+^ orthosilicate phosphors along with Ca^2+^ contents. Opt. Mater..

[26] Yun H., Park S. (2018). Blue-white-orange tunable Ba_6_Ca_3_YAlSi_6_O_24_:Eu^2+^, Mn^2+^ phosphors for NUV-pumped LEDs. Opt. Mater..

[27] Xia Z., Liu R.S., Huang K.W., Drozd V. (2012). Ca_2_Al_3_O_6_F:Eu^2+^: A green-emitting oxyfluoride phosphor for white light-emitting diodes. J. Mater. Chem..

[28] Liu C., Xia Z., Lian Z., Zhou J., Yan Q. (2013). Structure and luminescence properties of green-emitting NaBaScSi_2_O_7_:Eu^2+^ phosphors for near-UV-pumped light emitting diodes. J. Mater. Chem. C.

