# Supplementary figures and images for: Nonstoichiometric LaO0.65F1.7 Structure and Its Green Luminescence Property Doped with Bi3+ and Tb3+ Ions for Applying White UV LEDs
Source: Materials (Basel). 2022 Jun 14;15(12):4222. doi: 10.3390/ma15124222 (PMC9229386; doi:10.3390/ma15124222)

Figure S1. The plot of  $I_{SO}/I_S$  versus  $C_{Tb}^{a/3}$  ( $a = 6, 8, 10$ ).

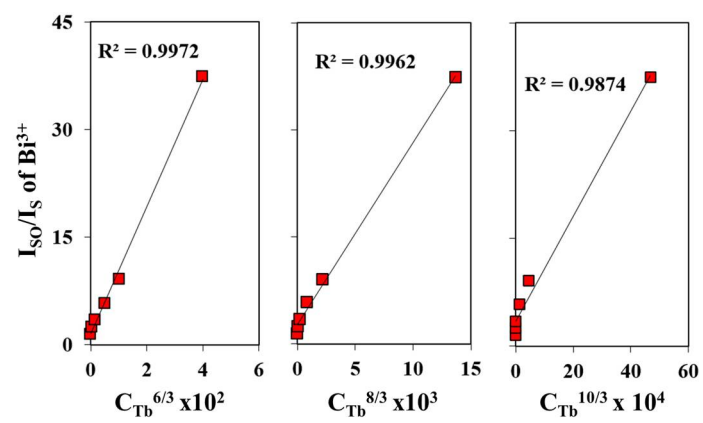

Supplement: Supplementary file 1 [file materials-15-04222-s001.zip › materials-1726854-supplementary.pdf]
